# The *Drosophila* ERG channel *seizure* plays a role in the neuronal homeostatic stress response

**DOI:** 10.1371/journal.pgen.1008288

**Published:** 2019-08-08

**Authors:** Alexis S. Hill, Poorva Jain, Nicole E. Folan, Yehuda Ben-Shahar

**Affiliations:** 1 Department of Biology, College of the Holy Cross, Worcester, Massachusetts, United States of America; 2 Department of Biology, Washington University in St. Louis, St. Louis, Missouri, United States of America; Katholieke Universiteit Leuven, BELGIUM

## Abstract

Neuronal physiology is particularly sensitive to acute stressors that affect excitability, many of which can trigger seizures and epilepsies. Although intrinsic neuronal homeostasis plays an important role in maintaining overall nervous system robustness and its resistance to stressors, the specific genetic and molecular mechanisms that underlie these processes are not well understood. Here we used a reverse genetic approach in *Drosophila* to test the hypothesis that specific voltage-gated ion channels contribute to neuronal homeostasis, robustness, and stress resistance. We found that the activity of the voltage-gated potassium channel *seizure* (*sei*), an ortholog of the mammalian ERG channel family, is essential for protecting flies from acute heat-induced seizures. Although *sei* is broadly expressed in the nervous system, our data indicate that its impact on the organismal robustness to acute environmental stress is primarily mediated via its action in excitatory neurons, the octopaminergic system, as well as neuropile ensheathing and perineurial glia. Furthermore, our studies suggest that human mutations in the human ERG channel (hERG), which have been primarily implicated in the cardiac Long QT Syndrome (LQTS), may also contribute to the high incidence of seizures in LQTS patients via a cardiovascular-independent neurogenic pathway.

## Author summary

Neurons are extremely sensitive to diverse environmental stressors, including rapid changes in the ambient temperature. To buffer environmental stress, many animals have evolved diverse physiological mechanisms to protect neuronal activity from acute and chronic stressors. Failures of these safeguards often lead to hyperexcitability, episodic seizures, and chronic epilepsy. However, although seizures and related syndromes are common, their underlying molecular and genetic factors, and their interactions with environmental triggers, remain mostly unknown. Here, we show that in the fruit fly, mutations in the ERG voltage-gated potassium channel *seizure* (*sei*), an ortholog of the human hERG channel that has been previously implicated in the cardiac Long-QT syndrome, also increases seizure susceptibility. We demonstrate that in addition to its cardiac expression, the *sei* channel is broadly expressed in the nervous system. In neurons, *sei* channels are enriched in axonal projections, and are specifically required in excitatory and octopaminergic modulatory neurons, as well as the non-neuronal glia, for maintaining organismal resistance to heat-induced seizures. Thus, our work indicates that the previously reported increase in seizure susceptibility in individuals with mutations in hERG is possibly related to its neuronal action, independent of its cardiac functions.

## Introduction

Neuronal homeostatic responses to acute and long-term environmental stressors are essential for maintaining robust behavioral outputs and overall organismal fitness [1–3]. Many environmental stressors, such as changes in temperature or oxygen availability, impact various aspects of neuronal system function [1]. Nervous systems must therefore compensate, in a homeostatic manner, in order to continue functioning in the presence of these stressors. At the neuronal level, the homeostatic response to stress depends on both synaptic and cell-intrinsic physiological processes that enable neurons to stably maintain optimal activity patterns [4–6]. The synaptic processes include both presynaptic mechanisms related to neurotransmitter release and postsynaptic mechanisms controlling neurotransmitter receptor localization, turnover, and control of downstream signaling pathways [2]. Previous theoretical and empirical studies in both invertebrate and mammalian species have suggested that neuronal intrinsic robustness depends on the expression and activity of specific combinations of ion channels and transporters, which can vary across neuronal cell types and individuals [7–10]. While some of the transcriptional and physiological processes that enable neurons to adjust their intrinsic activity levels in response to long-term stressors have been identified, primarily via the altered conductance of voltage-gated ion channels [11–13], most of the genetic and molecular mechanisms that mediate susceptibility to acute, environmentally-induced seizures, such as fever-induced febrile seizures, remain unknown [14–16].

In humans, seizures result from a diverse set of mechanisms that lead to an abnormal increase in electrical activity of the nervous system. A wide range of stressors have been associated with triggering seizures, including fevers, flickering lights, sleep deprivation, and emotional stress [17, 18]. A handful of genetic mutations have been linked to febrile [19–21] and photosensitive [22, 23] seizures, yet these only account for a small percentage of individuals experiencing seizures in response to these and other stressors.

Because of its small size, large surface-to-volume ratio, and its inability to internally regulate body temperature, the fruit fly *Drosophila melanogaster*, represents an excellent model for studying mechanisms underlying the neuronal response to acute heat stress [24]. To date, forward genetic screens in *Drosophila* have identified several mutations that lead to heat-induced seizures and paralysis [24–27]. These mutations seem to primarily affect the function of genes that encode voltage-gated sodium and potassium channels, and proteins associated with their neuronal function [28–31]. Here we tested the hypothesis that the knockdown of genes that are specifically important for the intrinsic neurophysiological homeostatic response to acute heat stress, would have little impact on fly behavior at permissive temperatures, but would lead to rapid paralysis under acute heat-stress conditions.

To test our hypothesis, we first employed a reverse genetic approach to identify candidate genes specifically involved in the neuronal homeostatic response to acute heat stress. By using a tissue-specific RNAi knockdown screen of voltage-gated potassium channels, we identified *seizure* (*sei*), the fly ortholog of the mammalian hERG channel (KCNH2) [26, 29, 32–35], as an essential element in the neuronal homeostatic response to acute heat stress. The *sei* gene was originally identified in a *Drosophila* forward mutagenesis screen for temperature-sensitive (ts) structural alleles of essential genes associated with neuronal excitability [33–35]. Although the original screen was designed to identify point-mutations that would lead to proteins that are functional at permissive temperature but would inactivate at non-permissive high temperature due to misfolding, we have recently shown that null alleles of *sei* and neuronal RNAi knockdown lead to high temperature-induced seizures, as in the original “ts” alleles [35]. These data indicate that the original *sei* alleles isolated were not true ts alleles. Instead, these data suggest that *sei* is not required for baseline neural excitability, but does play a role in the ability of neurons to maintain adaptive firing rates when exposed to acute heat stress. Furthermore, we have previously shown that temporal downregulation of *sei* expression specifically in neurons of the adult fly, or pharmacologically blocking SEI channel activity only in the adult stage, is sufficient to increase susceptibility to acute heat-induced seizures [35]. These data suggest that the impact of *sei* mutations on stress-induced seizures is primarily a consequence of physiological rather than developmental processes.

Previous studies have indicated that individuals who carry some of the dominant hERG mutations that cause the cardiac Long QT Syndrome (LQTS) [36, 37], often also suffer from high prevalence of generalized seizures [38, 39]. Yet, it is currently assumed that seizures in these patients represent a derived secondary outcome of the primary LQTS cardiac pathology [40–42]. However, the data presented here, as well as previous studies that showed that ERG channels are expressed in mammalian neuronal tissues [43, 44], and contribute to intrinsic spike frequency adaptation in cultured mouse neuroblastoma cells and cerebellar Purkinje neurons [45, 46], suggest that ERG channels also have a specific function within the nervous system.

By utilizing existing and novel genetic tools, here we show that the ERG channel *sei* is indeed essential for maintaining neuronal robustness under acute heat stress conditions in *Drosophila*. Specifically, we used an intersectional approach, combining the UAS-GAL4 and LexAOp-LexA binary transgene expression systems [47, 48], to show that *sei* is broadly expressed in the nervous system, in both neurons and glia. Yet, using RNAi to downregulate *sei* expression in specific cell types, we demonstrate that the contribution of *sei* to organismal behavioral resistance to acute heat stress is primarily mediated via its specific action in excitatory cholinergic and glutamatergic neurons, the octopaminergic system, as well as non-neuronal glia. Furthermore, by generating a CRISPR/cas9-derived GFP-tagged allele of the native *sei* locus, we also show that at the subcellular level, *sei* exerts its action primarily in axons and associated glia. Together, these studies indicate that mutations in hERG-like potassium channels may contribute directly to the etiology of stress-induced seizures in susceptible individuals by limiting the intrinsic neuronal homeostatic response to acute environmental stressors, possibly via homeostatic axonal spike frequency adaptation.

## Results

### Neuronal *sei* gene knockdown leads to acute heat-induced seizures

**Fig 1:**
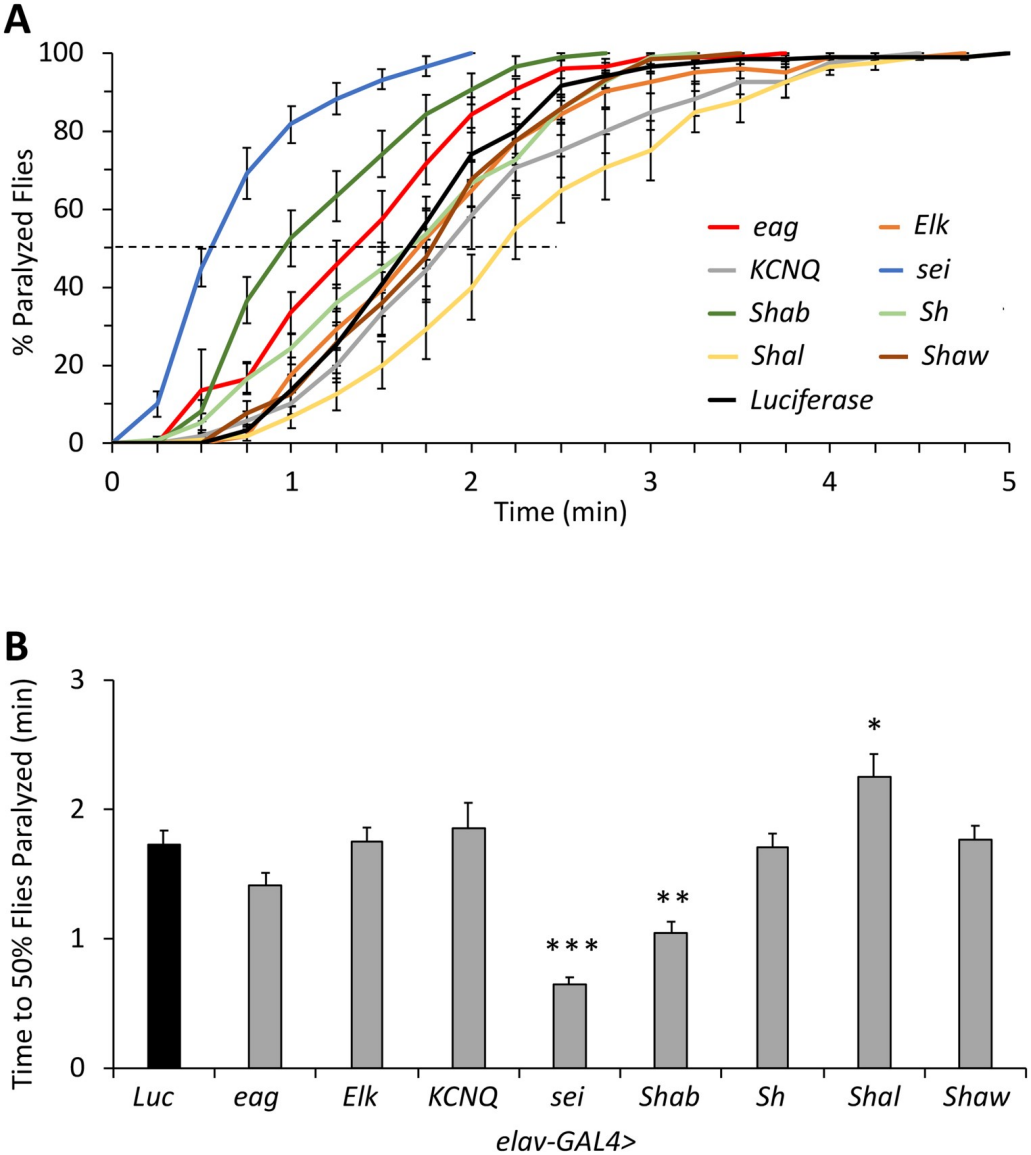
Neuronal (*elav-GAL4*) RNAi knockdown screen of *Drosophila* genes encoding voltage gated potassium channels. **A**) Cumulative percent paralyzed flies over time, with dotted line indicating 50% level. **B**) Direct comparison of time at which 50% of flies are paralyzed, using the same data as in A. n = 12 vials/genotype, 10 flies per vial, placed in 41–42°C water. ANOVA (*p* < 0.0001) followed by Dunnett’s *post hoc* test was used to determine groups significantly different than the *Luciferase* control. Data are presented as mean ±SEM, *p < 0.05, **p < 0.01, ***p < 0.001.

Previously published theoretical models and empirical studies have indicated that the actions of diverse voltage-gated potassium channels mediate action potential repolarization, and modulate the action potential threshold [49–52], which are important for the homeostatic regulation of synaptic activity and excitability [53–55]. Yet, which genes regulate the intrinsic capacity of neurons to buffer environmentally-induced hyperexcitability is mostly unknown. Thus, we initially hypothesized that the intrinsic ability of neurons to buffer acute heat stress is mediated, at least in part, by the action of specific voltage-gated potassium channels. Because most of these channels are expressed in both neuronal and non-neuronal tissues, we tested our hypothesis by using a neuronal-specific RNAi-dependent knockdown screen of all genes that encode voltage-gated potassium channels in the *Drosophila* genome. This screen revealed that the threshold to heat-induced seizures is lowered by neuronal knockdown of the genes *seizure* (*sei*) and *Shab*, and raised by neuronal knockdown of *Shal* (Fig 1A and 1B). These data suggest that different members of the Kv4-type voltage-gated potassium channels play diverse physiological roles in regulating the organismal susceptibility to acute heat stress.

**Fig 2:**
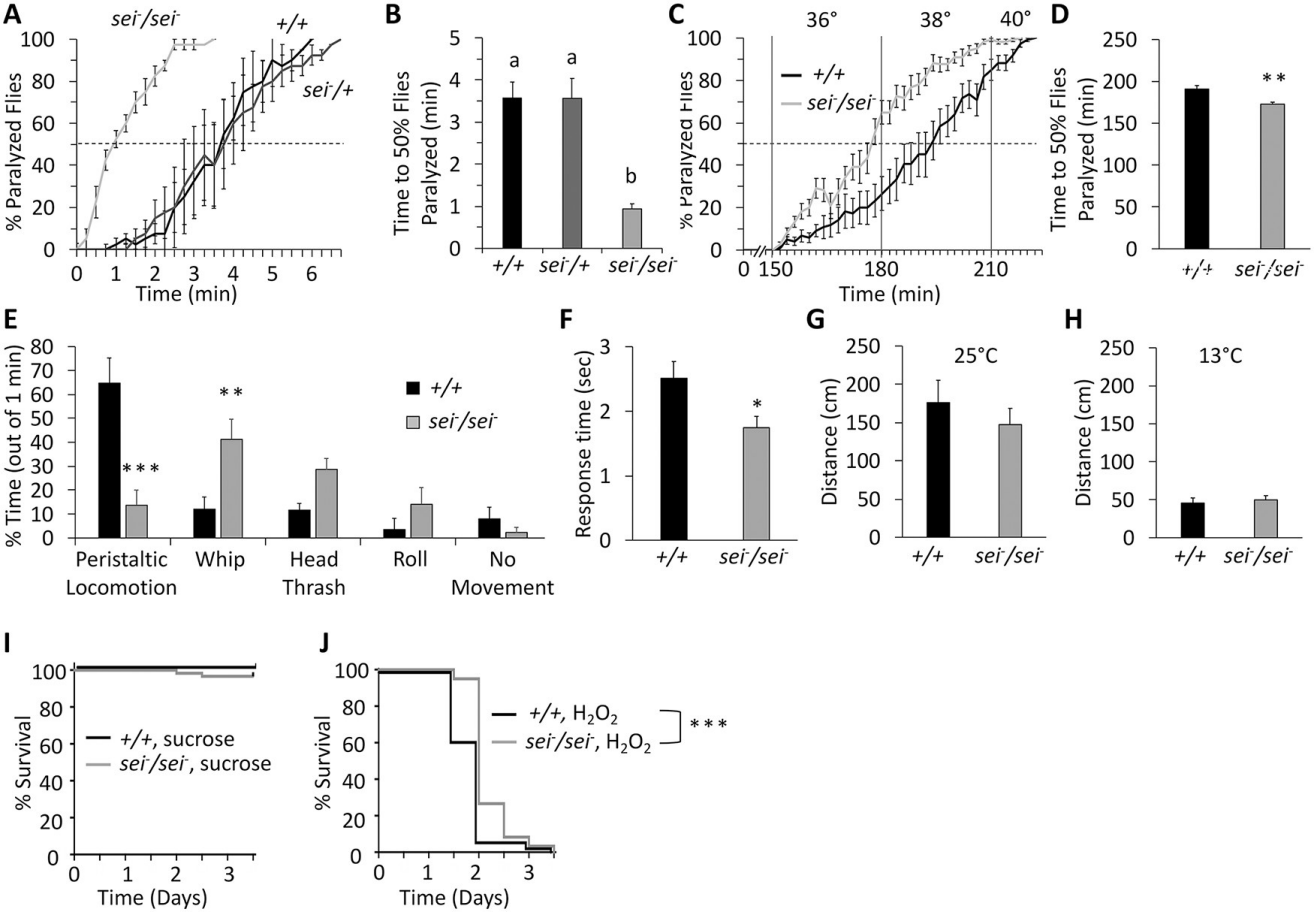
*sei* mutants display heat hypersensitivity. **A-B**) Acute heat assay paralysis behavior of homozygous and heterozygous *seiP* mutants and controls. n = 4 vials/genotype, 10 flies per vial, placed in 41–42°C water. Letters above bars represent significantly different groups by ANOVA (*p* < 0.001) followed by Tukey’s *post hoc* analysis (*p* < 0.01). **C-D**) Gradual heat assay paralysis behavior of *seiP* mutant flies and controls. n = 12 vials, 10 flies per vial. **E**) Larval behavioral responses when placed on an agar surface heated to 37 °C, in *seiP* mutants and controls. n = 9. **F**) Response time of *seiP* mutant larvae and controls to 50°C thermal nociception assay. **G-H**) *seiP* mutant and wildtype larvae distance travelled in five minute trials at room temperature (25°; n = 6) or cold temperature (13°; n = 12). **I-J**) Survival of adult male *seiP* mutants and controls exposed to sucrose alone or with H_2_O_2_ (n = 60 per group). Significance determined via log-rank (Mantel-Cox) test. Data are presented as mean ±SEM, *p < 0.05, **p < 0.01, ***p < 0.001 (Student’s *t*-test unless otherwise stated).

Because previous studies by us and others have shown that mutations in *sei* promote low neuronal and organismal resistance to acute heat stress [33–35], and *sei* had the strongest effect on lowering the threshold to heat-induced seizures in our screen (Fig 1A and 1B), we focused our following primary working hypotheses on the contribution of the *sei* channel to the intrinsic neuronal homeostatic response to acute environmental stress. Thus, we next used a null allele of *sei* [56, 57] to demonstrate that *sei* activity is specifically required for the ability of adult flies to resist the impact of acute heat stress (Fig 2A and 2B). In addition to its role in the neuronal response to acute heat stress, we also show that the *sei* mutation impaired the ability of flies to adapt to a gradual heat stress, where vials of flies were placed in an incubator, starting at room temperature (26°C) and increased 2°C every half hour (Fig 2C and 2D). Together, these data indicate that *sei* activity is not required for basal neuronal excitability, but is essential for the ability of neurons to maintain stable and adaptive firing rates under fluctuating environmental conditions.

In contrast to adult flies, which often have the ability to escape non-ideal environmental conditions, such as high temperatures, by flying, larvae are much more constrained. Thus, the protective role of *sei* might be more ecologically relevant to the pre-adult developmental stages. Our data indicate that, as in the adult, *sei* activity is also necessary for normal larval locomotion under acute heat stress conditions, as tested by placing larvae on a heated agar surface (Fig 2E). Furthermore, because larvae can sense acute nociceptive stimuli, such as heat, via the activity of their cuticular multidendritic (md) sensory neurons [58–60], we next tested the hypothesis that heat induced hyperexcitability in *sei* mutants would lead to nociception hypersensitivity. Indeed, we found that *sei* mutant larvae exhibit a significantly faster response when touched with a heated probe, relative to wild type control (Fig 2F), suggesting nociceptive system hypersensitivity, due to hyperexcitability of md sensory neurons, downstream circuits, or both. Together, these studies indicate that the *sei* channel plays an important role in maintaining neuronal stability and robustness, and protecting *Drosophila* neurons from environmentally-induced hyperexcitability.

Because previous investigations of the impact of temperature changes on neuronal activity have shown that neurons will respectively increase or decrease their firing rates in response to a rise or fall in ambient temperature [61–63], we next hypothesized that *sei* mutant flies might be protected from the effect of acute cold stress on neuronal activity. However, we found no effect of the *sei* mutation on larval locomotion on a 13°C cooled agar surface relative to wild type controls (Fig 2H). Thus, although the precise biophysical role of hERG-type voltage-gated potassium channels in regulating neuronal excitability remains elusive, the *in vivo* data presented here, as well as previously published *in vitro* studies [45, 46], indicate that hERG channels play a specific role in maintaining optimal neuronal activity by protecting neurons from environmentally-induced hyperexcitability but not hypoexcitability [35].

Next, we asked whether the susceptibility of *sei* mutants to heat stress represents a more general sensitivity to any environmental stressor. To answer this, we studied the effect of the *sei* mutation on survival during exposure to hydrogen peroxide (H_2_O_2_), a reactive oxygen species that induces oxidative stress, harming many molecular compounds and processes within cells [64, 65], and shown to lead to death in *Drosophila* [66, 67]. Surprisingly, we found that *sei* mutants actually exhibit higher resistance to the toxic effects of hydrogen peroxide relative to wild type controls (Fig 2J). While we do not yet know whether this phenotype is due to nervous system SEI function, nor the mechanism by which *sei* mutants have a slight resilience, these data nevertheless indicate that the impact of *sei* mutation in increasing susceptibility to heat stress is relatively specific, and does not generalize to all environmental stressors.

### Organismal resilience to heat stress requires the action of *sei* in excitatory and octopaminergic neurons, as well as glia

**Fig 3:**
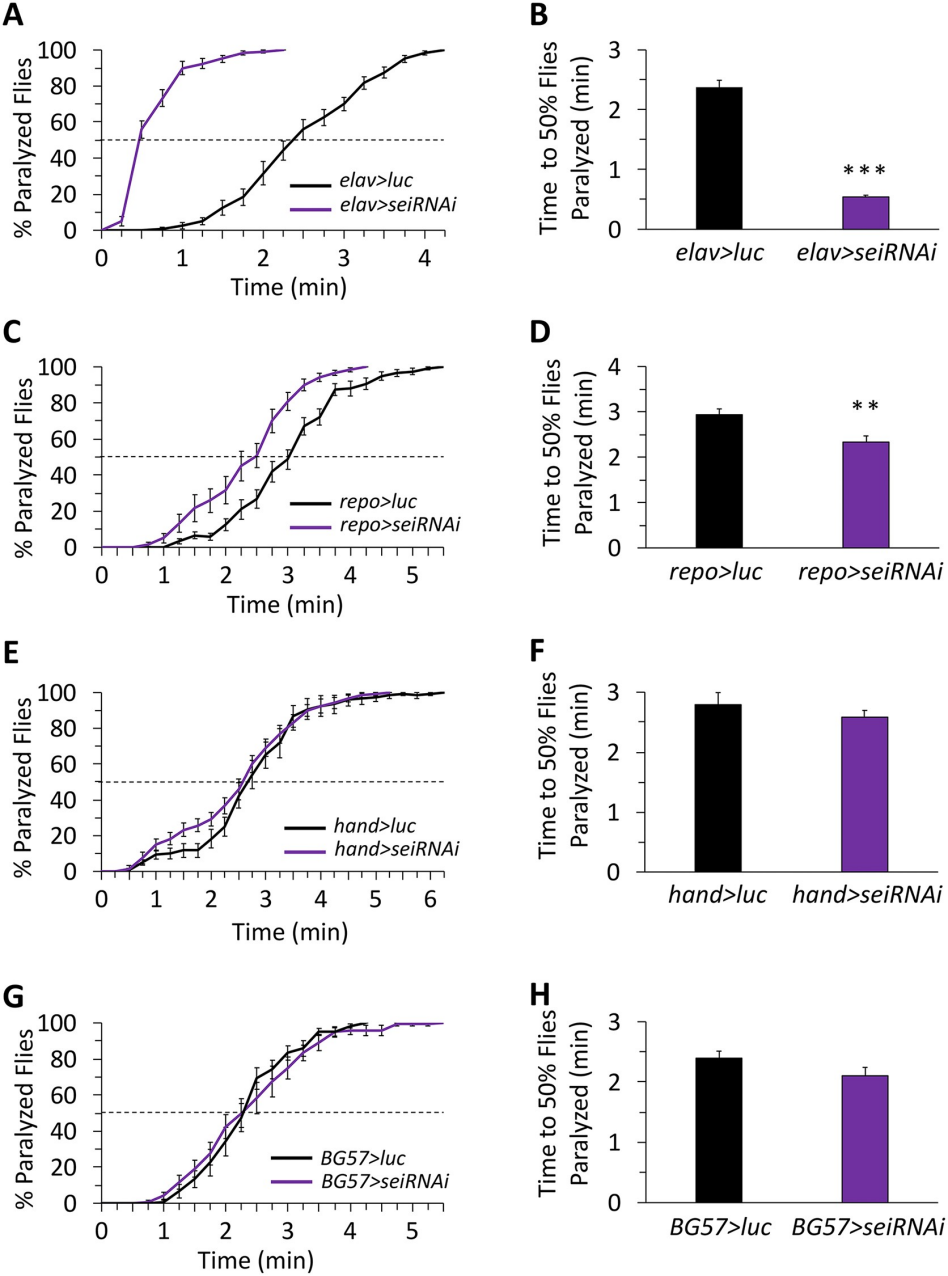
*sei* expression is required in neurons and glia for the homeostatic response to acute heat stress. A cell-type specific RNAi-dependent *sei* knockdown screen. **A-B**) Neurons (*elav-GAL4*); **C-D**) glia (*repo*-*GAL4*); **E-F**) heart (*hand-GAL4*); **G-H**) muscle (*BG57-GAL4*). n = 12 vials/genotype, 10 flies per vial, placed in 41–42°C water. Data are presented as mean ±SEM, **p < 0.01, ***p < 0.001 (Student’s *t*-test).

Previous studies by us and others have indicated that the *sei* gene is expressed in diverse neuronal and non-neuronal cell types, including cardiac and muscle cells [43, 68, 69], and that mutations in *sei* increase the overall organismal sensitivity to acute heat stress, resulting in shorter latency to heat-induced seizures and paralysis [33–35]. Yet, whether this organismal phenotype is driven by the action of *sei* in all cell types that express it was unknown. Therefore, we next used tissue-specific RNAi knockdown to determine which cell types require *sei* activity to protect animals from heat-induced seizures. Similarly to our previous work [35], we found that neuronal-specific knockdown of *sei* is sufficient to phenocopy the effect of the null allele on the susceptibility of adult flies to heat-induced seizures (Figs 1A, 1B, 3A and 3B). However, we were also surprised to find that, although not as striking as in the neuronal knockdown, the organismal response to heat stress also depends on the activity of *sei* in non-neuronal glia (Fig 3C and 3D). In contrast, although a previous study has indicated that *sei* activity is important for heart physiology [69], we found that *sei* knockdown in the heart or body muscles has no effect on seizure susceptibility (Fig 3E–3H). Together with the strong effect of pan-neuronal *sei* knockdown on heat-induced seizures, these data suggest that the effects of the *sei* mutations on seizure susceptibility are independent of *sei* action in the heart. Furthermore, the organismal resistance to acute heat-stress specifically depends on *sei* activity in the two primary cell types of the nervous system.

**Fig 4:**
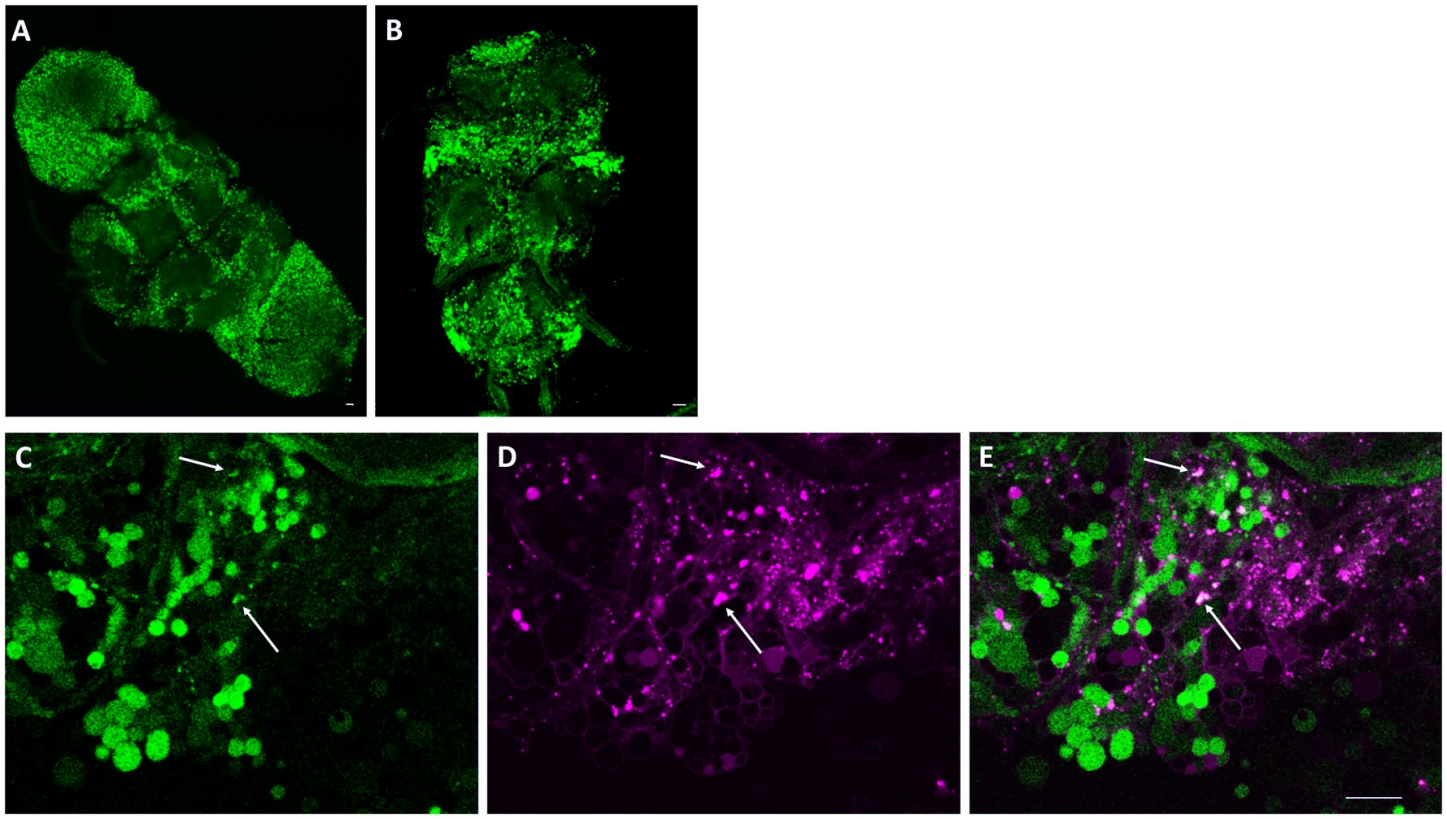
*sei* promoter drives expression in neurons and glia. **A-B**) Representative confocal z-stack images of an adult brain (**A**) and ventral nerve cord (**B**) from *sei-LexA>LexAOp-GFPnls* (green) flies. **C-E**) Overlap of *sei-LexA>LexAOp-GFPnls* (green) and glial marker *RepoGAL4>UAS-RedStinger* (magenta). Arrows point to examples of co-labeled cells. Scalebars are 20μm.

Because the fly CNS is a compact mosaic of different cell types, it is hard to establish whether *sei* is primarily expressed in neurons or glia. To address this, we generated a transgenic *Drosophila* line that expresses the *LexA* activator under the control of the putative *sei* promoter sequences [48, 70]. We then used this line to express a nuclear localized *EGFP* reporter (*GFPnls*) [71, 72], which indicated that *sei* is broadly expressed throughout the central nervous system (Fig 4A and 4B). To specifically identify *sei*-expressing glia, we next combined the *sei*-LexA line driving *GFPnls* with a red fluorescent protein *DsRed* including a nuclear localization signal (*RedStinger*) driven by the glia-specific *Repo-GAL4* line [73]. Confocal imaging of co-labeled brains showed that in addition to its broad neuronal expression pattern, *sei* is also expressed in a small fraction of brain glia (Fig 4C–4E, arrows). These data further indicate that the action of *sei* in the fly CNS is primarily mediated by its action in most neurons and some glia.

**Fig 5:**
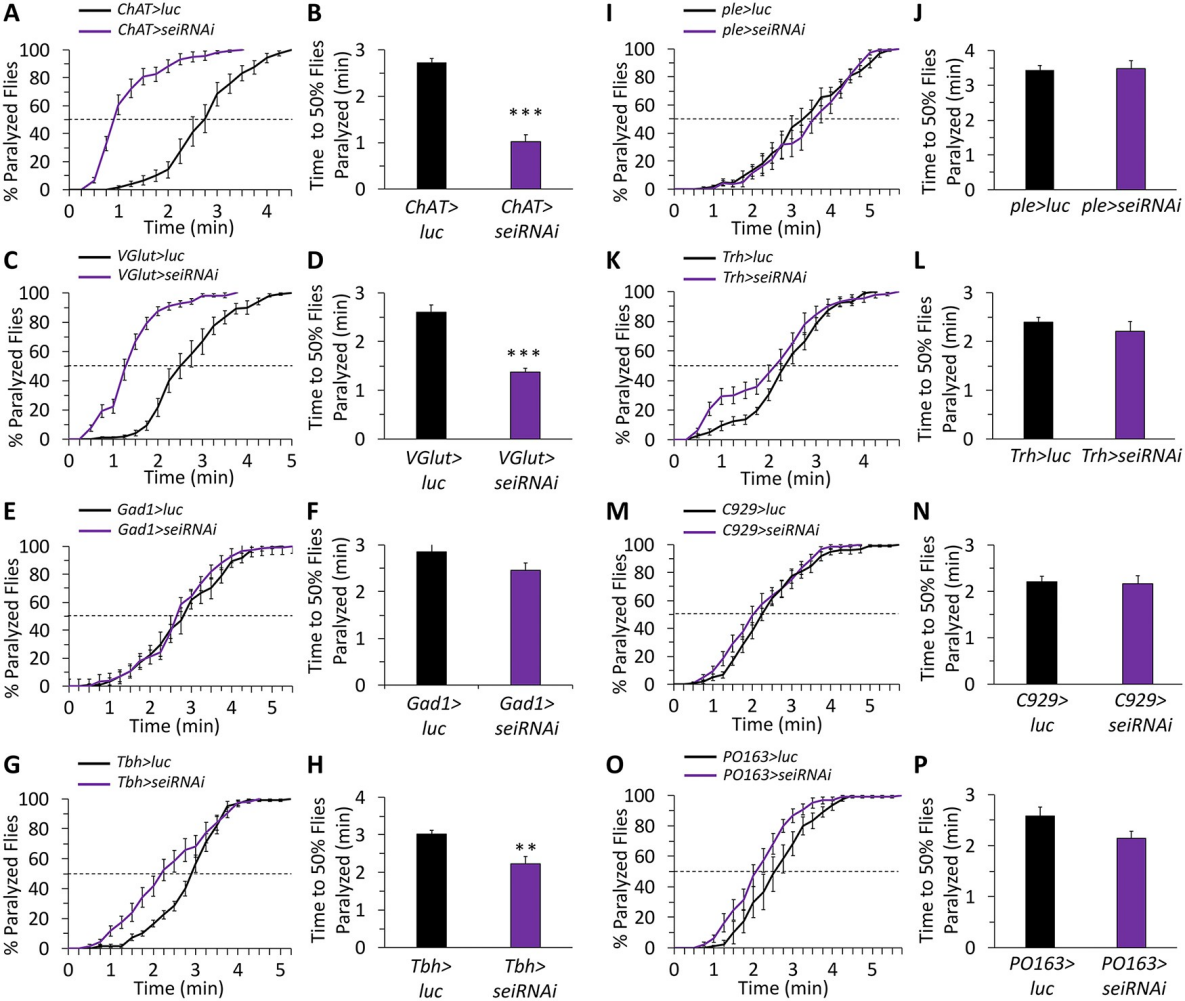
*sei* expression is specifically required in cholinergic, glutamatergic and octopaminergic neurons for response to acute heat stress. Neuronal subtype specific RNAi-dependent *sei* knockdown screen. **A-B**) Cholinergic neurons (*ChAT-GAL4*) n = 12 vials/genotype; **C-D**) Glutamatergic neurons (*VGlut-GAL4*) n = 6; **E-F**) GABAergic neurons (*Gad1-GAL4*) n = 12; **G-H**) Octopaminergic neurons (*Tbh-GAL4*) n = 12; **I-J**) Dopaminergic neurons (*ple-GAL4*) n = 12; **K-L**) Serotonergic neurons (*Trh-GAL4*) n = 12; **M-N**) Peptidergic neurons (*C929-GAL4*) n = 6; **O-P**) Sensory neurons (*PO163-GAL4*) n = 6. All experiments conducted with 10 flies per vial, placed in 41–42°C water. Data are presented as mean ±SEM, **p < 0.01, ***p < 0.001 (Student’s *t*-test).

Neurons are comprised of diverse cell types with different physiological properties and varying contributions to systems-level neural excitability. Therefore, we next wished to determine which neuronal subtypes might require *sei* activity for enabling the organismal response to acute heat stress. A broad screen of *sei* knockdown using several neuronal type-specific GAL4 driver lines revealed that the organismal response to heat stress depends on the expression of *sei* in cholinergic (Fig 5A and 5B) and glutamatergic (Fig 5C and 5D) excitatory neurons, but not in GABAergic inhibitory neurons (Fig 5E and 5F). We also observed a significant effect of *sei* knockdown in the modulatory octopaminergic system (Fig 5G and 5H) but not in the dopaminergic, serotonergic, peptidergic, or the peripheral sensory systems (Fig 5I–5P). Thus, our data suggest that *sei* plays an important role in protecting the nervous system from environmental stressors that could lead to general hyperexcitability and seizures by maintaining the neuronal robustness of excitatory and some neuromodulatory neurons.

**Fig 6:**
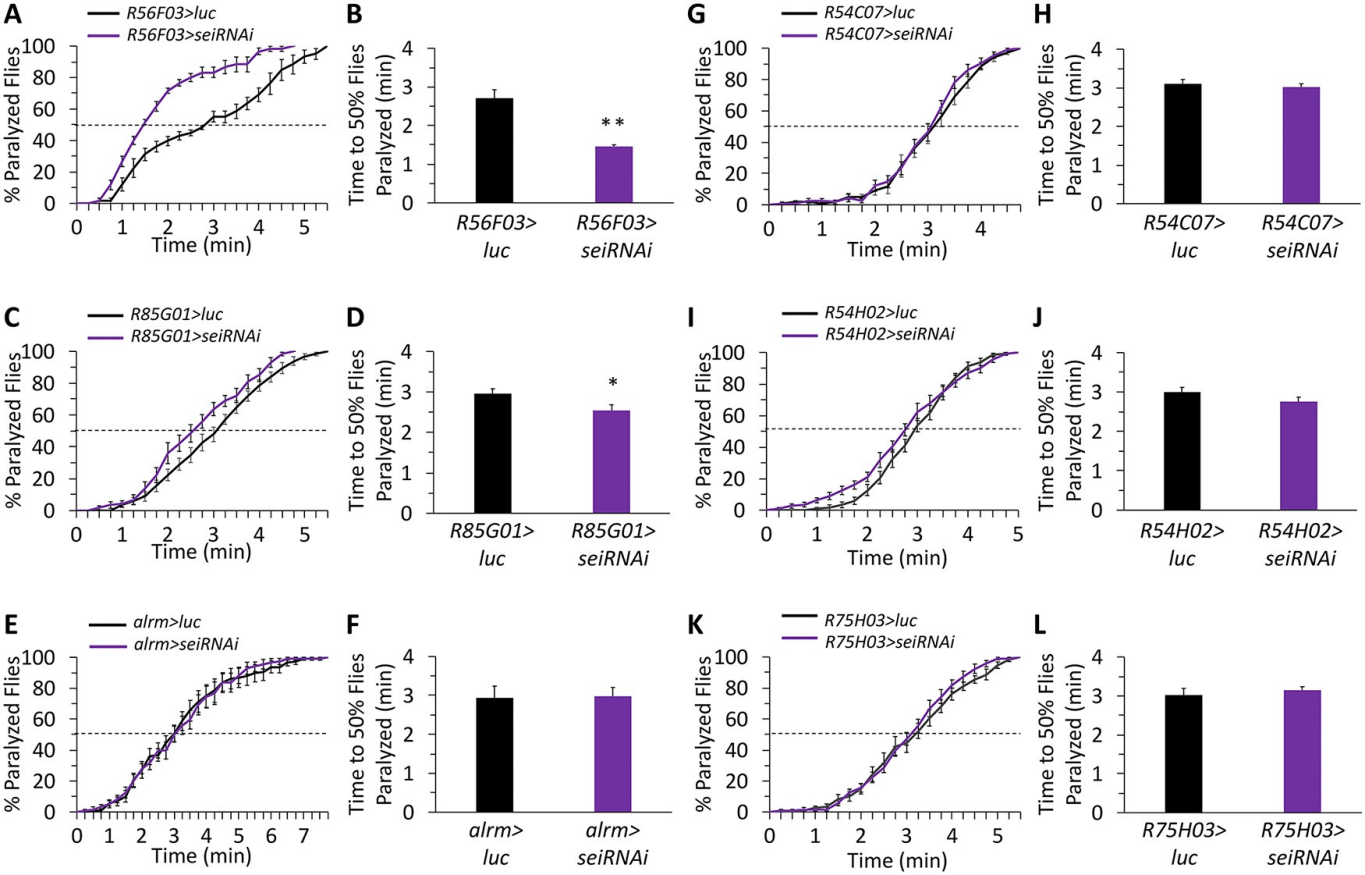
Organismal response to acute heat stress depends on *sei* action in neuropile ensheathing and perineurial glia. Glia subtype specific RNAi-dependent *sei* knockdown screen. **A-B**) Neuropile ensheathing glia (*R56F03-GAL4*) n = 6 vials/genotype; **C-D**) Perineurial glia (*R85G01-GAL4*) n = 12; **E-F**) Astrocyte-like glia (*alrm-GAL4*) n = 12; **G-H**) Subperineurial glia (*R54C07-GAL4*) n = 12; **I-J**) Cortex glia (*R54H02-GAL4*) n = 12; **K-L**) Tract ensheathing glia (*R75H03-GAL4*) n = 12. All experiments conducted with 10 flies per vial, placed in 41–42°C water. Data are presented as mean ±SEM, *p < 0.05, **p < 0.01 (Student’s *t*-test).

Similarly to neurons, *Drosophila* glia are comprised of several subtypes based on their localization, cellular morphology, and function [74, 75]. Therefore, we also wished to determine which glia require *sei* expression for the organismal response to acute heat stress. By screening a collection of recently published glia subtype-specific GAL4 lines [75], we found that the response to acute heat stress specifically depends on *sei* action in neuropile ensheathing glia (Fig 6A and 6B), and to a lesser extent in perineurial glia (Fig 6C and 6D), but not in astrocyte-like, subperineurial, cortex or tract ensheathing glia (Fig 6E–6L). Although the contribution of SEI channel activity to specific glia subtypes remains unknown, these data suggest that hERG-like potassium currents in some glia play an important role in maintaining organismal robustness to some acute environmental stressors.

### Mutations in *sei* lead to a subtle increase in synaptic arborization at the larval, but not adult, NMJ

**Fig 7:**
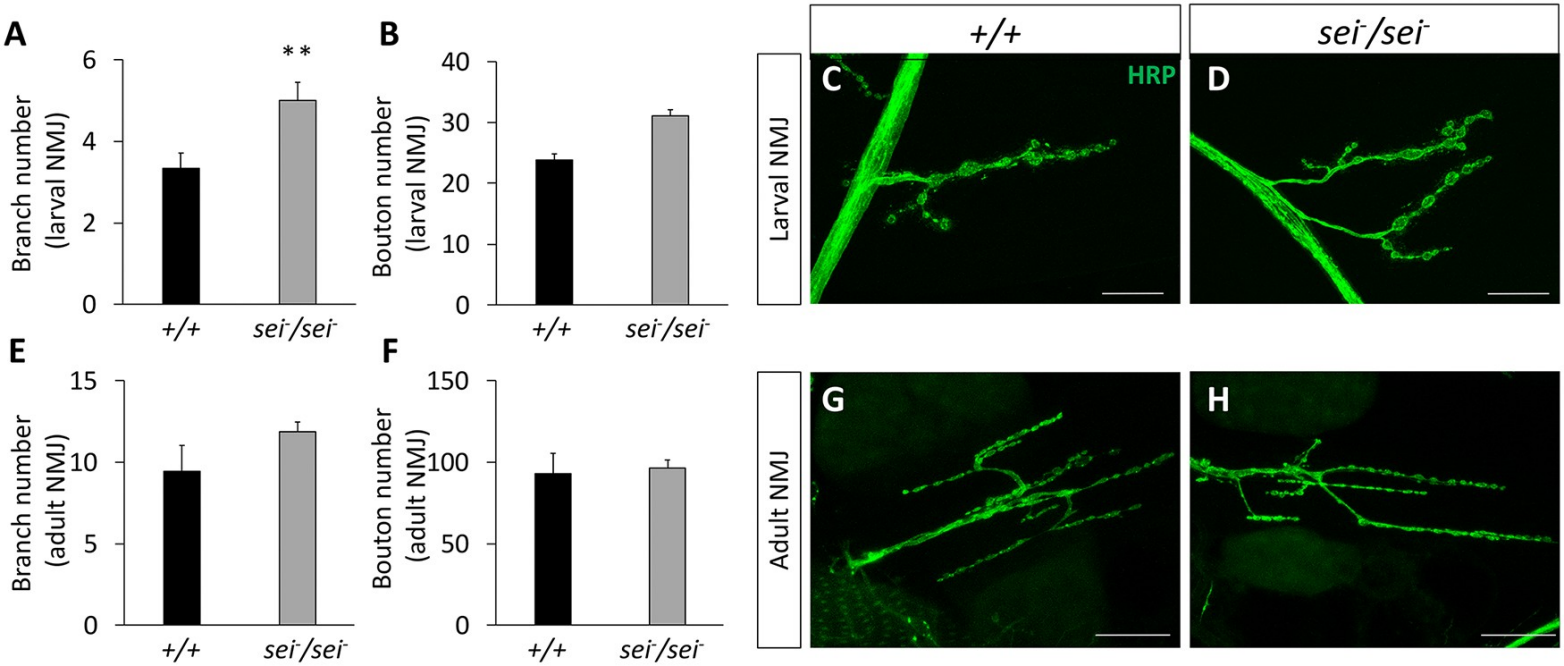
Effects of *sei* mutation on NMJ synaptic morphology in larvae and adults. **A**) *SeiP* mutants display increased branching at the larval neuromuscular junction (NMJ). n = 11–12. **B**) No difference in the number of boutons was observed between *seiP* mutants and controls at the larval NMJ. n = 11-12/genotype. **C-D**) Representative 63x confocal z-stack images of larval NMJs stained with anti-HRP antibody labeling neuronal processes. **E-F**) No differences were observed in number of branches or bouton number at the NMJ of adult ventral abdominal muscles. n = 7/genotype. **G-H**) Representative 63x confocal z-stack images of adult abdominal NMJs stained with anti-HRP antibody labeling neuronal processes. Data are presented as mean ±SEM, **p < 0.01 (Student’s *t*-test). Scale bars are 20μm.

Previous studies have shown that in cultured mammalian neurons, ERG-related channels contribute to the regulation of action potential firing rate via spike frequency adaptation, a cell-intrinsic process [45, 46]. These data suggest that *sei* plays a similar role in fly neurons, which likely explains the heat-induced hyperexcitability and rapid seizure development observed in *sei* mutant animals. Nonetheless, a previous study suggested that mutations in *sei* can also lead to a mild increase in axonal branches and boutons at the larval neuromuscular junction (NMJ) [76], which suggests that the observed mutant phenotype may be also driven, at least in part, via synaptic mechanisms. Therefore, we next examined the synaptic morphology of the larval and adult NMJs. We found that although the *sei* mutation does lead to an increase in the number of branches at the larval NMJ (Fig 7A), it has no effect on synaptic bouton numbers (Fig 7B). Furthermore, we observed no effects of the *sei* mutation on either synaptic branches or bouton numbers in the NMJs of the adult ventral abdominal muscles (Fig 7E and 7F). Together, these data indicate that the effects of *sei* mutations on heat-induced seizures in adult flies are primarily mediated via its intrinsic physiological action in neurons rather than via processes associated with synaptic development.

### SEI channels are localized to axonal processes

**Fig 8:**
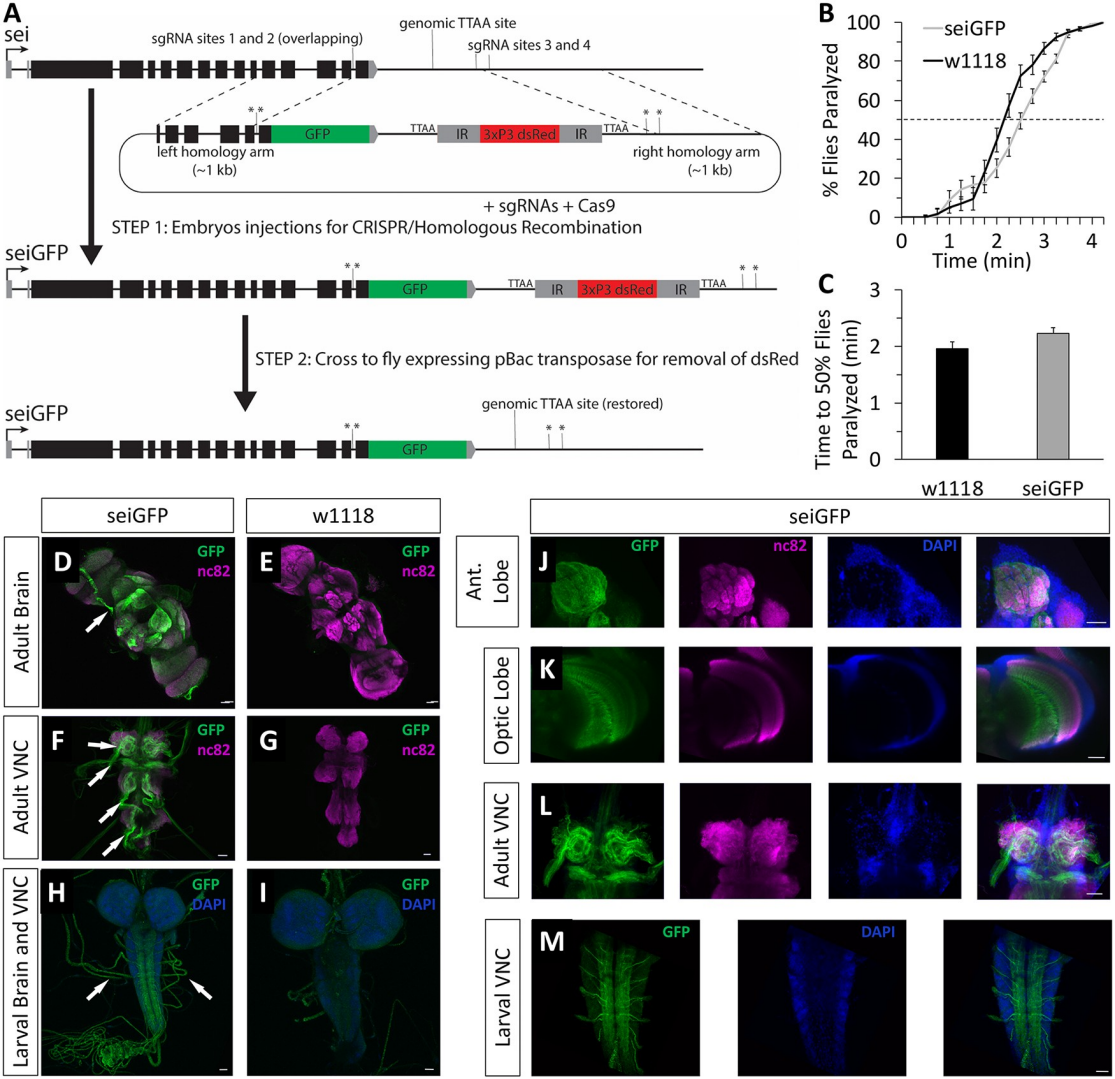
Nervous system expression of *seiGFP* in adults and larvae is primarily localized to neuronal axons. **A**) Strategy for the generation of the *seiGFP* allele by using CRISPR/*Cas9*-dependent DNA editing. Stars represent single nucleotide substitutions in the PAMs of sgRNA sites. **B-C**) Behavior of *seiGFP* and wildtype flies in the acute heat assay. n = 12. Data was analyzed using Student’s *t*-test and presented as mean ±SEM. **D-G**) Representative 20x confocal z-stack images of the adult brain (**D-E**) and adult ventral nerve cord (**F-G**) following immunostaining for *seiGFP* (green) and nc82 (magenta). **H-I**) 20x confocal z-stack images of the larval brain and ventral nerve cord of *seiGFP* (green) and DAPI (blue). Note that low levels of green autofluorescence are observed in the w^1118^ larval brain. **J-M**) 40x confocal z-stack images of the adult antennal lobe (**J**), optic lobe (**K**) and ventral nerve cord (**L**) with *seiGFP* (green), nc82 (magenta) and DAPI (blue). **M**) 40x confocal z-stack images of the larval ventral nerve cord of *seiGFP* (green) and DAPI (blue). Scalebars are 20μm. White arrows point to neuronal tracks with enriched *seiGFP* localization.

The subcellular localization of various voltage gated ion channels plays an important role in determining how they might be contributing to neuronal signaling and excitability [77–79]. For example, ion channels localized to axons generally impact action potential generation, propagation, and modulation, while those localized to dendrites influence integration of synaptic inputs, propagation of electrical activity to the soma, and action potential backpropagation [77, 80, 81]. Nevertheless, ion channels with specific subcellular enrichment in either dendrites, cell bodies, axons, or presynaptic terminals have all been implicated in human epilepsies [82]. Therefore, we next determined the subcellular localization of native SEI channels by generating a C-terminus GFP-tagged allele of the endogenous *sei* locus (Fig 8A). We found that the response of homozygous *seiGFP* flies to heat stress is not different from wild type animals, which indicates that the tagged protein forms wild-type like channels (Fig 8B and 8C). We next used an anti-GFP antibody to probe the subcellular spatial distribution of *seiGFP* channels in the larval and adult nervous systems. These studies revealed that SEI is primarily localized to the axonal membranes of most neurons, but not in dendrites or somas, as visualized by the lack of colocalization between GFP and the nuclear stain DAPI (Fig 8D–8M), and the enriched *sei* localization to sensory axonal tracks in the adult brain (Fig 8D), thoracic ganglion (Fig 8F), and motor and sensory neuron axons in larvae (Fig 8H). While the glial function of SEI channels remains unknown, the enrichment of SEI channels in axons supports a model whereby ERG channels contribute to the intrinsic homeostatic regulation of optimal neuronal activity via the modulation of action potentials. This model is further supported by the previously reported influence of mammalian ERG channels on spike frequency adaptation in cultured mammalian neurons [45, 46].

## Discussion

Previous theoretical and empirical studies of neural circuit adaptability, and by extension, the ability of animals to maintain robust and adaptive behavioral outputs in unstable environments, depends on both the intrinsic homeostatic capacity of neurons to maintain an optimal activity pattern, and the ability of neural circuits to maintain stable outputs via the homeostatic regulation of neuronal connectivity and synaptic activity [4–6]. Yet, despite its high incidence, the majority of genetic and molecular factors that regulate neuronal homeostasis, and increase susceptibility to seizures, remain mostly unknown [14–16]. Here, we show that the *Drosophila* voltage-gated potassium channels *sei*, *Shab*, and *Shal* impact the neuronal homeostatic response to acute heat stress. Furthermore, we show that the organismal capacity to buffer the effects of acute heat stress depends on the independent activity of *sei* in both neurons and glia. We also found that although *sei* is broadly expressed in the nervous system, its contribution to the overall organismal resistance to acute heat stress seems to be specifically driven by its action in cholinergic and glutamatergic excitatory neurons, and neuropile-ensheathing glia, as well as to a lesser extent in the modulatory octopaminergic system and perineurial glia. In addition, we developed genetic tools to show that SEI is expressed in the axons of neurons and in glia. Together, our data highlight the important role of *sei* in the organismal homeostatic response to acute environmental stress, by providing robustness to both the intrinsic activity of specific neuronal populations, and the neural circuits that harbor them. We expect that future work on other voltage gated potassium channel genes, and well as other ion channels and transporters, in both neurons and glia, will continue to shed light on the genetic programs that control robust and homeostatic processes within the nervous system.

How ERG-type potassium channels might contribute to neuronal intrinsic homeostasis during bouts of acute stress is not well understood. Nonetheless, *in vivo* and *in vitro* studies in *Drosophila* and mammalian models have suggested that ERG channels have little effect on baseline neuronal firing rate, but can prevent rapid firing in response to environmental or electrophysiological stimuli that induce hyperexcitability [35, 45, 46]. We have previously shown that in *Drosophila* motor neurons, basal neuronal firing patterns are unaffected by the *sei* mutation at optimal 25°C, but become hyperexcitable in response to a rapid temperature increase [35]. Similarly, in electrophysiological studies of mammalian brain slices, *in vitro* cultured neurons, and heterologously-expressed mammalian ERG channels, pharmacological blockers of hERG channels have little effect on firing rates in response to small current injections, but greatly diminish spike frequency adaptation in response to large current injections, resulting in rapid firing rates [45, 46]. The presence of SEI channels specifically in axons (Fig 8) suggests that they do not affect the propagation of dendritic potentials, but rather limit the rate of action potential generation and propagation, which is sufficient to prevent rapid firing rates. Together, these data suggest a model whereby ERG-like potassium channels play a crucial role in mediating the neuronal homeostatic response to acute stress by protecting neurons from rapid increase in firing rates, and therefore, support neuronal robustness when exposed to extreme environmental fluctuations.

At the neuronal network level, seizures are thought to result from an imbalance between excitatory and inhibitory neural signaling pathways [15, 83]. We found that knocking down *sei* specifically in all cholinergic neurons, the primary excitatory pathway in the fly central nervous system, is sufficient to phenocopy the effects of *sei* null mutations on the organismal resistance to heat-induced seizures. These results are similar to previous studies, which showed that increasing activity of the cholinergic system in flies, via genetic manipulations of voltage gated sodium channels and optogenetic neural activation, is sufficient to increase seizure-related and paralytic behavior [84–87]. The simplest interpretation of these data together is that the lack of *sei* in cholinergic excitatory neurons makes them hypersensitive to heat-induced hyperexcitability, which subsequently surpasses the buffering capacity of the inhibitory neurotransmission pathways, and therefore leads to the rapid development of generalized seizures and paralysis.

Additionally, we observed a large increase in seizure susceptibility when *sei* is knocked down in glutamatergic neurons. Although we and others have previously demonstrated that the activity of motor neurons, which in insects are primarily glutamatergic, is increased in seizure-susceptible mutant flies [84, 88], this finding suggests that the decreased intrinsic ability of motor neurons to resist acute stress is sufficient for inducing organismal seizure-like phenotype (Fig 5C and 5D). Nevertheless, we currently cannot exclude the possibility that the observed effect of knocking down *sei* expression in glutamatergic neurons on organismal sensitivity to heat stress is mediated via the action of a small number of modulatory glutamatergic neurons within the central nervous system [89].

We also observed an impairment in the organismal homeostatic response to acute heat stress when *sei* is specifically knocked-down in the modulatory octopaminergic system (Fig 5G and 5H). Previous studies of the octopaminergic system in *Drosophila* and other insects have indicated that octopamine and related biogenic amines have broad impact on diverse neuronal processes at the developmental and physiological timescales [90, 91]. Because *sei* mutant flies seem to have normal behaviors when housed under constant optimal conditions, it is likely that the effects of knocking down *sei* in octopaminergic neurons on heat-induced seizures are physiological, not developmental. Although we currently do not know which specific elements of the octopaminergic system play a role in the organismal response to acute heat stress, previous work has shown that exogenous application of octopamine in *Drosophila* increases contraction force of muscles and their response to synaptically driven contractions [92]. Therefore, one possible mechanism by which *sei* knockdown in octopaminergic neurons might affect observed heat-induced seizures is via the direct modulation of the neuromuscular junction. Octopamine has also been shown to play important roles in the central nervous system, including modulation of behaviors related to motivation, sleep, aggression, social behaviors and learning and memory [90, 93–96]. Therefore, *sei* knockdown in octopaminergic neurons may result in a broader shift in synaptic processes associated with the homeostatic maintenance of the balance between excitatory and inhibitory pathways under acute heat stress conditions.

The important role of *sei* activity in regulating the capacity of the nervous system to buffer acute environmental stress is further supported by our discovery that its knockdown in glia also increased susceptibility to acute heat-induced seizures (Figs 3C, 3D and 6). These data are in agreement with previously published studies, which demonstrated that the knockdown of genes associated with ionic homeostasis in glia can increase seizure susceptibility in *Drosophila* [97–101]. Previous studies have suggested that some glia are important for maintaining synaptic activity and homeostasis, and that disrupting glia functions could contribute to the etiology of seizures because of their role in modulating extracellular potassium concentration, adenosine levels, the size of the extracellular space, and uptake of neurotransmitters [97, 102–106]. Of all the glia, our data indicate that *sei* is specifically important in neuropile-ensheathing glia. A complete picture of the specific functions of neuropile ensheathing glia has yet to emerge, yet studies manipulating genes in this cell type have implicated roles in phagocytosis of injured neurons [107], organization of neural circuits [108], and glutamate metabolism [109]. However, how the action of voltage gated ion channels such as *sei* in glia might affect these specific processes remains mostly unknown. Nevertheless, glial expression of another voltage gated potassium channel that is associated with human epilepsy, KCNJ10, has been shown to lead to epileptic activity in a mouse model, possibly via its role in buffering extracellular potassium and glutamate [110, 111]. Whether hERG-like channels play a similar role in glia remains to be explored.

Together, the data we present here provide important insights into the possible role of hERG channels in regulating neuronal robustness and susceptibility to stress-induced seizures. From a clinical perspective, our data suggest that the high incidence of generalized seizures that has been reported in LQTS patients that carry mutations in the hERG genes [38] might not be a secondary cardiogenic comorbidity, as is currently often assumed [40–42]. Instead, because about 40% of patients that carry LQTS-related hERG mutations have reported a personal history of seizures, as compared to less than 20% in LQTS patients with similar cardiac pathologies that are due to mutations in other genes [38], we hypothesize that seizure etiology in many LQTS patients is likely due to the direct impact of mutations in hERG on nervous system functions, independent of their cardiovascular condition. Therefore, we predict that it is possible that some unidentified mutations in hERG might be causally related to epilepsies, independent of the presentation of any LQTS-related pathologies, and may represent novel genetic risk factors for seizures.

The studies we describe here provide compelling evidence that hERG channels play an essential role in protecting the nervous system from acute environmental stressors, such as heat, which could potentially lead to hyperexcitability and seizures. Furthermore, we show that in *Drosophila*, the activity of the ERG channel *sei* contributes to neuronal and behavioral robustness via its action in independent cell types in the nervous system. These important insights should help us to better understand how the nervous system responds to acute environmental stressors, and possibly provide important mechanistic insights into some of the known pathologies associated with hERG mutations in human patients.

## Materials and methods

### Fly stocks and genetics

Flies (*Drosophila melanogaster*) were raised on standard corn syrup-soy food (Archon Scientific) at 25°C temperature, 70% humidity, on a 12:12 light/dark cycle. Unless specifically noted, wild type control line used was *w*^*1118*^. All fly strains were either produced in the Ben-Shahar lab or obtained from the Bloomington Stock Center (stock numbers in parentheses). UAS-RNAi TRiP lines [112] used in the initial screen included *sei* (#31681), *shab* (#25805), *eag* (#31678), *shaker* (#53347), *shaw* (#28346), *shal* (#31879), *elk* (#25821) and *kcnq* (#27252). The TRiP UAS-*Luciferase* RNAi was used a control (#35789), and UAS-RNAi lines were driven by the *elav-GAL4; UAS-Dicer2* line (#25750) (Fig 1A and 1B). For the cell-type-specific *sei* knockdown screen (Figs 3, 5 and 6), the UAS-RNAi for *sei* and *luciferase* were each recombined with *UAS-Dcr2*. The phenotypic assessment of these RNAi lines indicates that the observed effects were specific to some GAL4 lines but not all, suggesting that the UAS-RNAi transgene alone has no effect on *sei* expression. The original null *seiP* allele from Bloomington (#21935) was backcrossed for 6 generations into the *w*^*1118*^ wild type strain (Bloomington #6326) [113]. Other transgenic lines from the Bloomington stock center included: *UAS-RedStinger* (#8546), *LexAOp-GFPnls* (#29954), *elav-GAL4* [114] (#458), *Repo-GAL4* (#7415), *hand-GAL4* (#48396), *ChAT-GAL4* (#6798), *VGlut-GAL4* (#60312), *Gad1-GAL4* (#51630), *ple-GAL4* (#8848), *Tbh-GAL4* (#39939), *Trh-GAL4* (#49258), and *C929-GAL4* (#25373). *BG57-GAL4* [115] and *PO163-GAL4* were from the Dickman (USC) and Zlatic (HHMI Janelia Research Campus) labs respectively. The following GAL4 lines were used for glia subtype-specific expression [75]: neuropile ensheathing, *R56F03-GAL4* (#39157); tract ensheathing, *R75H03-GAL4* (#39908); perineurial, *R85G01-GAL4* (#40436); subperineurial, *R54C07-GAL4* (#50472); cortex, *R54H02-GAL4* (#45784); astrocyte-like, *alrm-GAL4* (#67032).

The C-terminus GFP-tagged allele of *sei* was generated via CRISPR/*Cas9-*dependent editing by using a modified “scarless” strategy (www.flyCRISPR.molbio.wisc.edu)[116, 117]. Specifically, four sgRNAs TGTAAGCGAATACCACGTTG, GACAGCATTCTCCCGCAACG, GAAGCAGAAGCAGGTAACTC, AGGTGAGTGAGTTACTCATC, which flank the targeted genomic *sei* sequence were designed using flyRNAi.org/crispr. Complementary oligos that correspond to each individual sgRNA (IDT) were cloned into the pDCC6 plasmid (a gift from Peter Duchek, Addgene plasmid # 59985), which also includes the coding sequence for *Cas9* [118], by using the BbsI restriction enzyme (NEB). The donor plasmid for homologous recombination was constructed by using a Golden Gate assembly [119] to recombine four DNA elements: 1) A backbone with ampicillin resistance (pBS-GGAC-ATGC plasmid, a gift from Frank Schnorrer, Addgene #60949)[120]; 2) Eye-specific dsRed reporter driven by the 3XP3 promoter, flanked by two *PiggyBac* transposase recognition sites, which was PCR-amplified from the pHD-ScalessDsRed plasmid (a gift from Kate O’Connor-Giles, Drosophila Genome Resource Center #1364) using the following primers 5’-CACACCACGTCTCATTAACCCTAGAAAGATAATCATATTGTG-3’ and 5’- CACACCACGTCTCACCCTAGAAAGATAGTCTGCGT-3’ (the primers included BsmBI restriction enzyme sites and overhangs corresponding to the left and right homology arms); 3–4) The left and right homology arms, which consisted of 1kb genomic DNA fragments upstream and downstream of the sgRNA sites respectively. Single base pair mutations were introduced into the PAM sequence at each sgRNA binding site on the homology arms to prevent Cas9-dependent cutting of the donor plasmid. The right homology arm also included the GFP coding sequence in frame with the 3’ end of the last coding exon of *sei*, immediately upstream of the endogenous stop codon (Fig 8A). pDCC6 plasmids containing sgRNA and cas9 sequences (100 ng/μL) and the donor plasmid (500 ng/μL) were co-injected into *y*^-^*w*^-^ background. Subsequently, correct genomic integration of the GFP tag was verified by screening for DsRed-positive animals, followed by sequencing of genomic PCR fragments. The final tagged *sei* allele was generated by removing the DsRed cassette via the introduction of the *piggyBac* transposase (Bloomington #8285) (Fig 8A).

The *sei-LexA* transgenic flies were generated by amplifying a 2612 bp genomic DNA fragment upstream of the *sei* start codon by using the following PCR primers: 5’-GTCGACCGCCGGCAAAGTATCAACAT-3’ and 5’-GCGGCCGCTTTTAAGTCTGCAAAGTATAGAAACG-3’, followed by cloning into the pENTR1A plasmid (ThermoFisher) with SalI and NotI restriction enzymes. The *sei* promoter fragment was then recombined into the pBPnlsLexA::p65Uw vector (a gift from Gerald Rubin, Addgene plasmid # 26230)[48] by using the Gateway reaction (ThermoFisher). The *sei-LexA* containing plasmid was integrated into the fly genome by using a line carrying a PhiC31 integrase landing site on Chromosome III (Bloomington #24483)[121].

### Behavioral response to acute heat stress

Assays were performed as previously described [35]. In short, two-day old flies were collected and transferred into standard vials containing food (five per sex in each vial). On the following day, flies were flipped into an empty vial, and tested within the next hour. For testing, vials with 10 flies were individually submerged into a 41–42°C water bath and observed for seizure-like behavior and paralysis. The cumulative number of paralyzed flies (immobile at the bottom of the vial) was recorded every 15 seconds. The time at which 50% of the flies in a given vial were paralyzed was used as a measure of seizure susceptibility for statistical analysis.

### Behavioral response to gradual heat stress

Two-day old flies were housed in groups of 10 as above. Subsequently, vials were placed in a temperature-controlled incubator (Fisher Scientific Isotemp) with a glass door, which allowed continuous video recording of their behavior. To test for the ability of flies to adapt to gradual temperature increase, flies were first acclimated to 26°C followed by a 2°C increase every 30 minutes to a maximum of 42°C. The number of paralyzed flies was recorded every two minutes.

### Larval locomotion behavior

A 60mm plastic petri dish was filled with 3% agar, and placed on a Peltier plate surface of a PCR machine, which was set to either 37°C or 13°C for the heat or cold stress respectively. Identical tests at 25°C were used as controls. For the heat stress condition, locomotion was assayed by placing individual third instar foraging larvae on the agar surface and video recording (Logitech C920 Webcam) for one minute. Larva locomotion was analyzed by assessing the amount of time spent executing the following specific behaviors: peristaltic locomotion, whipping, head thrashing, rolling and no movement [122](S1 Video). For the cold stress condition, individual larvae were allowed to acclimate for 30s before their behavior was recorded for four min. Behaviors of all larvae were tracked and analyzed by using a custom designed motion tracker system [123], which enabled computer-assisted analyses of distance traveled.

### Larval nociceptive response to heat

Foraging 3^rd^ instar larvae were removed from bottles, washed in water, and placed on a water-saturated 3% agar plate. Behavioral tests were conducted in constant 25°C and 70% humidity. Larvae were allowed to acclimate to the agar plate for 10 seconds. A custom-made heat probe (Thermal Solutions Controls and Indicators Corporation) set to 50°C was used to gently touch the side of the larva’s body, and the amount of time for the larva to roll over was recorded. For each genotype, 47–50 larvae were individually tested.

### Hydrogen peroxide treatment

At seven days of age, male flies were transferred into empty standard fly vials (Genesee Scientific), housed ten per vial. Daily, 900 uL of 1% sucrose solution, with or without 3% hydrogen peroxide (H_2_O_2_), was added to a Kimwipe packed tightly at the bottom of each vial. Survival was assessed every 12 hours.

### Immunostaining and imaging

Analyses of NMJ morphology were done as previously described [124, 125]. In short, animals were dissected to expose larval body wall muscles or adult ventral abdominal muscles. Samples were pinned on sylgard plates, fixed in 4% PFA for 20 minutes, washed with PBST+0.1% Triton-X (PBST), and then blocked with Superblock (ThermoFisher) for one hour. Samples were then incubated with FITC-conjugated goat anti-HRP (123-095-021, Jackson ImmunoResearch) to label neurons, diluted 1:1000 in Superblock, for 1 hour at room temperature. Samples were then washed in PBST, mounted using vectashield (Vector Laboratories), and imaged using a laser scanning confocal microscope (Leica TCS SP5).

Third-instar larval brains were dissected and then fixed, washed and blocked as above. Brains were subsequently incubated overnight at 4°C with rabbit anti-GFP (A-11122, ThermoFisher) diluted in Superblock at 1:1000. After washing with PBST, larval brains were incubated for 2 hours at room temperature with secondary antibody Alexa Fluor 488-conjugated goat anti-rabbit (A-11034, ThermoFisher) and FITC-conjugated goat anti-HRP (123-095-021, Jackson ImmunoResearch) to label neurons, each diluted 1:1000 in Superblock. After secondary incubation, brains were washed in PBST, mounted using FluoroGel II with DAPI (ThermoFisher), and imaged using an laser scanning confocal microscope (Nikon A1Si).

Adult brains were dissected, fixed and blocked as above, then subsequently incubated overnight at 4°C in rabbit anti-GFP (A-11122, ThermoFisher) diluted at 1:1000 and mouse anti-*Brp* (NC82; Developmental Studies Hybridoma Bank) diluted 1:33 in Superblock. After washing with PBST, adult brains were incubated overnight at 4°C with secondary antibodies Alexa Fluor 488-conjugated anti-rabbit (A-11034, ThermoFisher) and Alexa Fluor rhodamine-conjugated donkey anti-mouse (sc-2300, Santa Cruz Biotechnology), each diluted 1:500 in Superblock. Adult brains were mounted and imaged as above. To image live brains expressing either *RedStinger* or *GFPnls* nuclear markers, the tissues were dissected, mounted in PBS, and imaged on a confocal microscope within one hour of the dissection.

### Statistical analysis

Indicated statistical comparisons were analyzed by using Excel (Microsoft) and Prism 7 (GraphPad). Statistical significance was set at *p*<0.05.

## Supporting information

S1 Video Heat induced *seiP* mutant larval behavior. (MP4)
